# Emergence and Evolution of Unique Plasmids Harboring *bla*_IMP-70_ and *bla*_CTX-M-253_ in Multidrug-Resistant Providencia rettgeri

**DOI:** 10.1128/spectrum.01204-22

**Published:** 2022-07-06

**Authors:** Mako Watanabe, Ryuichi Nakano, Ayako Tanouchi, Akiyo Nakano, Yuki Suzuki, Kai Saito, Ryuji Sakata, Miho Ogawa, Hisakazu Yano

**Affiliations:** a Department of Microbiology and Infectious Diseases, Nara Medical University, Kashihara, Nara, Japan; b BML, Inc., Kawagoe, Saitama, Japan; Universidad de Buenos Aires, Facultad de Farmacia y Bioquímica

**Keywords:** *Providencia rettgeri*, multidrug-resistant Enterobacterales, β-lactamase, IMP-70, CTX-M-253, plasmid

## Abstract

Although the prevalence of carbapenem-resistant Enterobacterales remains low in Japan, these bacteria are a growing problem worldwide, owing to their multidrug resistance phenotype. We isolated a multidrug-resistant Providencia rettgeri strain, NR1418, harboring a rare *bla*_IMP_ variant, *bla*_IMP-70_, a novel *bla*_CTX-M_ variant, designated *bla*_CTX-M-253_, and *bla*_MOX-1_. This strain is resistant to β-lactams, amikacin, levofloxacin, and colistin. Genomic analysis revealed that NR1418 carries two plasmids, designated pNR1418-1 and pNR1418-2. The pNR1418-1 plasmid harbors *bla*_CTX-M-253_, *bla*_TEM-1_, and *bla*_MOX-1_, while the pNR1418-2 plasmid harbors *bla*_IMP-70_, which is located in a class 1 integron. Both plasmids exhibit high similarities with the plasmid of the *P. rettgeri* isolate BML2526, which also harbors *bla*_IMP-70_ and was identified in the same region of Japan as NR1418 at a different point in time. This indicates the possibility of the emergence and evolution of IMP-70-producing *P. rettgeri* and suggests that the plasmid of BML2526 may have occurred following recombination of the two plasmids harbored by NR1418. Further, *bla*_IMP-70_ and *bla*_CTX-M-253_ were found on unique plasmids, indicating that they likely evolved through mutations and recombination.

**IMPORTANCE** Although Providencia rettgeri is an opportunistic pathogen, its intrinsic resistance to colistin and tigecycline makes the treatment of carbapenem-resistant *P. rettgeri* challenging. We isolated a multidrug-resistant *P. rettgeri* strain which harbored a rare *bla*_IMP_ variant, *bla*_IMP-70_, a novel *bla*_CTX-M_ variant, *bla*_CTX-M-253_, and *bla*_MOX-1_ from a urinary sample obtained in Osaka, Japan. We investigated its genetic structure and evaluated the evolution of the plasmids carrying these genes. We show that *bla*_IMP-70_, *bla*_CTX-M-253_, and *bla*_MOX-1_ are present on unique plasmids and that they have high similarities to the plasmid of another IMP-70-producing *P. rettgeri* isolate that was identified as being from the same location. The evolution of plasmids through mutations and recombination may play a role in the development and spread of multidrug resistance.

## OBSERVATION

Providencia rettgeri, a member of Enterobacterales, is an emerging opportunistic pathogen that is often associated with urinary tract infections. The spread of carbapenem-resistant Enterobacterales (CRE) is a growing problem worldwide, as they exhibit multidrug resistance. *P. rettgeri* is usually susceptible to most antibiotics; however, it is intrinsically resistant to colistin and tigecycline, making the treatment of carbapenem-resistant *P. rettgeri* challenging. Recently, carbapenem-resistant *P. rettgeri* has been reported in several countries (1–3). NDM-1-positive *P. rettgeri* plays a major role in the spread of antibiotic resistance in Latin America (4). In Japan, the prevalence of CRE remains low (0.31% in 2020) (5), and the IMP-type metallo-β-lactamase is the most common carbapenemase (6). Although carbapenem-resistant *P. rettgeri* has been infrequently reported, there are a few reports of IMP-producing *P. rettgeri* (7–9). In this study, we isolated a multidrug-resistant *P. rettgeri* strain, NR1418, harboring a rare *bla*_IMP_ variant, *bla*_IMP-70_, which has only been reported in Japan (9). This isolate also carries a novel *bla*_CTX-M_ variant, *bla*_CTX-M-253_, and *bla*_MOX-1_. We further investigated the genetic structure of *P. rettgeri* coharboring *bla*_IMP-70_, *bla*_CTX-M-253_, and *bla*_MOX-1_ using genomic analysis and evaluated the evolution of the plasmids carrying these genes.

NR1418 was isolated from a urinary sample from a catheterized patient at a hospital in Osaka, Japan, in 2015. NR1418 was identified as *P. rettgeri* using matrix-assisted laser desorption ionization time-of-flight mass spectrometry (VITEK MS). Antimicrobial susceptibilities to antibiotics, excluding colistin, were determined using the agar dilution method; the susceptibility to colistin was determined using the broth microdilution method, according to the guidelines of the Clinical and Laboratory Standards Institute (10). NR1418 was resistant to all antibiotics tested, including β-lactams, amikacin, levofloxacin, and colistin (Table 1). The results of the carbapenemase inactivation method (11, 12) and the modified carbapenemase inactivation method (13) were positive, indicating that NR1418 produces carbapenemase.

**TABLE 1:** Susceptibilities of Providencia rettgeri NR1418 harboring *bla*_IMP-70_, *bla*_CTX-M-253_, *bla*_TEM-1_, and *bla*_MOX-1_ and its transformants

Strains	β-Lactamase genes	MIC (μg/mL)^*a*^													
		PIP	TZP	CPD	CTX	CTX+CLA	CAZ	FEP	CMZ	ATM	IPM	MEM	LVX	AMK	CST
*P. rettgeri* NR1418	*bla*_IMP-70_, *bla*_CTX-M-253_, *bla*_MOX-1_, *bla*_TEM-1_	>256	32	>256	>256	>256	>256	>256	>256	32	>256	>256	32	256	>256
E. coli pNR1418-1/TOP10	*bla*_CTX-M-253_, *bla*_MOX-1_, *bla*_TEM-1_	>256	4	256	128	16	8	2	16	4	0.25	≤0.06	≤0.06	2	0.5
E. coli pNR1418-2/TOP10	*bla*_IMP-70_	2	2	>256	128	64	>256	32	128	≤0.06	4	8	≤0.06	32	0.25
E. coli TOP10		2	2	1	≤0.06	≤0.06	0.25	≤0.06	1	≤0.06	0.25	≤0.06	≤0.06	2	0.5

a Antibiotics: PIP, piperacillin; TZP, piperacillin-tazobactam; CPD, cefpodoxime; CTX, cefotaxime; CLA, clavulanic acid; CAZ, ceftazidime; FEP, cefepime; CMZ, cefmetazole; ATM, aztreonam; IPM, imipenem; MEM, meropenem; LVX, levofloxacin; AMK, amikacin; CST, colistin.

β-Lactamase genes were identified using PCR and Sanger sequencing (14, 15), which revealed that NR1418 harbors *bla*_IMP-70_, *bla*_TEM-1_, *bla*_MOX-1_, and a novel CTX-M β-lactamase gene, *bla*_CTX-M-253_ (GenBank accession no. LC670768). CTX-M-253 is identical to *bla*_CTX-M-2_, except for a single amino acid substitution, Ala80Val. This substitution is associated with higher structural stability and catalytic activity in CTX-M-55, which is a CTX-M-15 variant resulting from the same substitution (16). The role of Ala80Val in increasing resistance or stability has been reported for other β-lactamases, as well (17, 18), and the substitution may play a role in CTX-M-253. However, further investigation is needed to determine the actual changes in the structure and activity of CTX-M-253 compared to those of CTX-M-2. IMP-70 (GenBank accession no. LC348383) is an IMP variant that we registered in 2017. IMP-70 differs from IMP-10 by a single amino acid substitution, Phe69Val, and from IMP-1 by an additional substitution, Val49Phe.

The transferability of the β-lactamase genes was tested using filter mating experiments with Escherichia coli J53 as the recipient strain (19). However, the genes were not transferred by conjugation. Instead, they were introduced into E. coli TOP10 (Thermo Fisher Scientific, Waltham, MA, USA) by transformation. Two types of transformants were obtained. One transformant harbored *bla*_CTX-M-253_, *bla*_TEM-1_, and *bla*_MOX-1_ (pNR1418-1/TOP10), while the other transformant harbored *bla*_IMP-70_ (pNR1418-2/TOP10). It was also found that pNR1418-1/TOP10 was resistant to piperacillin, cefotaxime, and cefpodoxime and was inhibited by clavulanic acid. Further, pNR1418-2/TOP10 was resistant to cephems and carbapenems.

The whole-genome of NR1418 was sequenced using MiSeq (Illumina) and MinION (Oxford Nanopore), and a hybrid *de novo* assembly was performed with Unicycler v0.4.8 (20). The genome sequences were annotated using DFAST v1.5.0 (21) and corrected manually using BLAST (https://blast.ncbi.nlm.nih.gov/Blast.cgi). Resistance genes were analyzed using ResFinder 4.1 (22). The assembled genome consisted of three circular contigs: a 4,371,793 bp chromosome, a 172,709 bp plasmid, pNR1418-1 (GenBank accession no. AP025670), and a 128,012 bp plasmid, pNR1418-2 (GenBank accession no. AP025671). The average depth was 260x. An average nucleotide identity (ANI) analysis performed using JSpeciesWS (23) with P. rettgeri DSM 1131 (GenBank accession no. ACCI02000000), *P. rettgeri* Dmel1 (GenBank accession no. AJSB01000000), *P. rustigianii* NCTC11802 (GenBank accession no. UGTY01000000), P. alcalifaciens 205/92 (GenBank accession no. JALD01000000), and P. stuartii INSRA21868 (GenBank accession no. LGYB01000000) as reference genomes showed ANI values (ANIb) of 98.48, 91.23, 77.55, 77.49, and 77.21%, respectively. Thus, NR1418 was identified as *P. rettgeri* through ANI analysis, as well. The identification of plasmid replicons using PlasmidFinder (24) showed that pNR1418-1 hosts IncC and IncT replicons, whereas pNR1418-2 harbors a col3M replicon.

**FIG 1:**
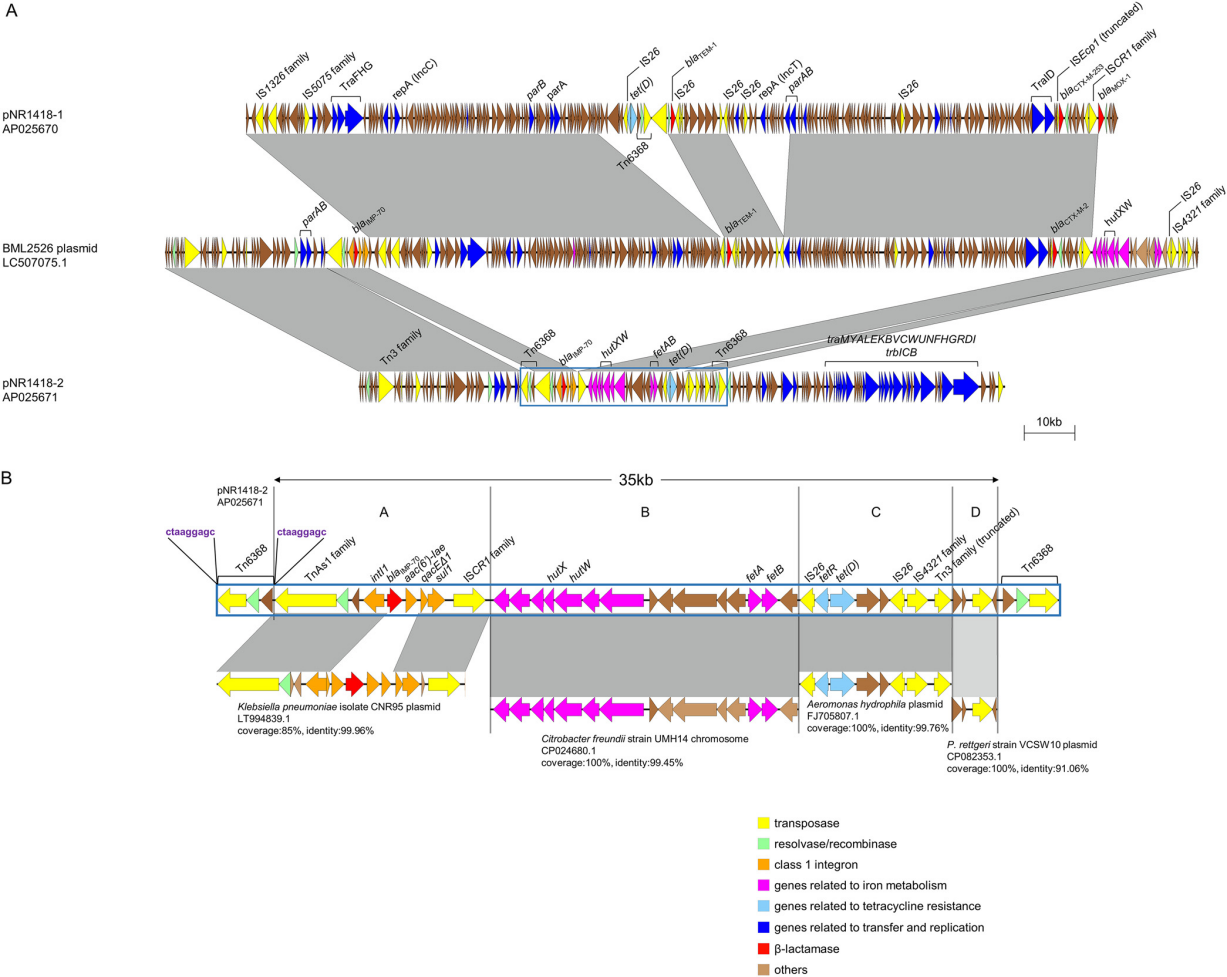
Genetic environments of plasmids harbored by IMP-70-producing Providencia rettgeri. (A) BLAST comparison of pNR1418-1, pNR1418-2, and the plasmid of BML2526. The comparisons were visualized with Easyfig version 2.2.2 (25). The plasmid of BML2526 consists of regions identical to parts of pNR1418-1 and pNR1418-2. Dark gray bars indicate nucleotide identity >99% in a window of 3000 bp. The 35 kb region flanked by Tn6368 in pNR1418-2 is shown inside a blue box, and its genetic structures are described in panel B. (B) Schematic representation of the 35 kb region flanked by Tn6368 in pNR1418-2. Structures with high similarity to each region are shown in the bottom row. Region A is identical to a structure identified in a plasmid of a Klebsiella pneumoniae isolate, except for the gene cassettes in the class 1 integron. Structures highly similar to regions B through D can be found in other strains. Transposases are seen at the border of every region. The 9 bp TSD pattern flanking Tn6368 is shown in purple. Dark and light gray bars indicate nucleotide identity >99% and >90%, respectively, in a window of 1000 bp.

The BLAST analysis showed that pNR1418-1 harbors *bla*_CTX-M-253_, *bla*_TEM-1_, and *bla*_MOX-1_ (Fig. 1A). A truncated IS*Ecp1* was found upstream of *bla*_CTX-M-253_, and *bla*_MOX-1_ was located 6.9 kb downstream. The pNR1418-2 plasmid harbors *bla*_IMP-70_, located in a class 1 integron structure along with *aac(6’)-Iae*, encoding aminoglycoside resistance. Both pNR1418-1 and pNR1418-2 have tra genes, and pNR1418-2 also has trb genes; however, these plasmids were not transferred by conjugation in this study. Further study is needed to determine if other factors, such as the compatibility of the recipient strain and the plasmids, may be involved. Both pNR1418-1 and pNR1418-2 had high similarities with the plasmid of the *P. rettgeri* isolate BML2526, which harbors *bla*_IMP-70_ (9). BML2526 harbors *bla*_IMP-70_, *bla*_CTX-M-2_, and *bla*_TEM-1_, but it does not harbor *bla*_MOX-1_, and *bla*_IMP-70_ is located in the same integron as pNR1418-2. In addition, the structure surrounding *bla*_CTX-M-2_ is similar to that surrounding *bla*_CTX-M-253_, indicating that *bla*_CTX-M-253_ may have occurred as a result of a point mutation in *bla*_CTX-M-2._

The integron containing *bla*_IMP-70_ in pNR1418-2 is located within a region flanked by two copies of Tn6368. This approximately 35 kb region could be divided further into four regions according to its similarity with other reported structures (Fig. 1B). Region A includes the class 1 integron containing *bla*_IMP-70_ and has a Tn3-like element TnAs1 family transposase and an IS*91*-like element IS*CR1* family transposase, at either end. Region B mostly consists of genes involved in iron metabolism and shares 99.45% identity with the chromosome of Citrobacter freundii strain UMH14 (GenBank accession no. CP024680.1). Region C has a tetracycline resistance gene, *tet*(D), as well as its transcriptional regulator, *tetR*, flanked by two copies of IS*26* and followed by an IS*4321* family transposase and a truncated Tn3 family transposase. Region D consists mostly of hypothetical proteins and shows the highest similarity to the *P. rettgeri* strain VCSW10 plasmid pVCSW10.2 (GenBank accession no. CP082353.1), with a shared identity of 91.06%. The remaining region of pNR1418-2 shows the highest similarity to the Pectobacterium carotovorum plasmid Drgb2 (GenBank accession no. KT351733.1), with a coverage of 62% and a shared identity of 96.97%. Analysis of target site duplication (TSD) patterns revealed a 9 bp TSD pattern (ctaaggagc) flanking Tn6368, located next to region A. As transposases of transposons and insertion elements are located at the border of each region, it is possible that the accumulation of several regions via transposable elements resulted in pNR1418-2. An exact match of region A was found in the plasmid of BML2526; however, an approximately 140 kb region similar to pNR1418-1 exists between *sul1* and the IS*CR1* family transposase. Regions similar to regions B and D follow; however, some parts of region C are missing, except for IS*26*, the IS*4321* family transposase, and the truncated Tn3 family transposase. The region 18 kb upstream of region A is also identical to a region in BML2526. No copies of Tn6368 were found on the plasmid of BML2526. Interestingly, NR1418 and BML2526 were identified in Osaka, Japan, at different points in time; NR1418 was isolated in 2015, and BML2526 was isolated in 2018, indicating the possibility of the emergence and evolution of IMP-70-producing *P. rettgeri*. These findings suggest that the plasmid of BML2526 may have occurred following recombination of the two plasmids harbored by NR1418.

In conclusion, we characterized a multidrug-resistant *P. rettgeri* strain carrying multiple β-lactamases, including *bla*_IMP-70_, *bla*_TEM-1_, *bla*_MOX-1_, and *bla*_CTX-M-253_. These β-lactamases were found on unique plasmids, indicating that they likely evolved through mutations and recombination. Some NDM-1-positive plasmids harbored by *P. rettgeri* likely originated by a cointegration of plasmids, making them easier to disseminate among Enterobacteriaceae (4). These findings suggest that the cointegration of plasmids in *P. rettgeri* might not be unusual and that they may play a role in the transmission of clinically relevant β-lactamases. Although *P. rettgeri* is not a common clinical pathogen, we highlight its potential role in the spread of multidrug resistance and the importance of continued monitoring and surveillance of IMP-producing *P. rettgeri*.

### Data availability.

The assembled nucleotide sequences of NR1418 have been deposited in GenBank under the accession numbers AP025669–AP025671.
